# Multi-target chimaeric VLP as a therapeutic vaccine in a model of colorectal cancer

**DOI:** 10.1186/s40425-017-0270-1

**Published:** 2017-08-15

**Authors:** Braeden Donaldson, Farah Al-Barwani, Simon J. Pelham, Katie Young, Vernon K. Ward, Sarah L. Young

**Affiliations:** 1 Department of Microbiology and Immunology, School of Biomedical Sciences, University of Otago, Dunedin, New Zealand; 2 Department of Pathology, Dunedin School of Medicine, University of Otago, PO Box 56, Dunedin, 9054 New Zealand

**Keywords:** Colorectal cancer, Virus-like particles, RHDV, Therapeutic vaccine, CpG

## Abstract

**Background:**

Colorectal cancer is responsible for almost 700,000 deaths annually worldwide. Therapeutic vaccination is a promising alternative to conventional treatment for colorectal cancer, using vaccines to induce targeted immune responses against tumour-associated antigens. In this study, we have developed chimaeric virus-like particles (VLP), a form of non-infectious non-replicative subunit vaccine consisting of rabbit haemorrhagic disease virus (RHDV) VP60 capsid proteins containing recombinantly inserted epitopes from murine topoisomerase IIα and survivin. These vaccines were developed in mono- (T.VP60, S.VP60) and multi-target (TS.VP60) forms, aiming to elucidate the potential benefits from multi-target vaccination.

**Methods:**

Chimaeric RHDV VLP were developed by recombinantly inserting immune epitopes at the N-terminus of VP60. Vaccines were tested against a murine model of colorectal cancer by establishing MC38-OVA tumours subcutaneously. Unmethylated CpG DNA oligonucleotides (CpGs) were used as a vaccine adjuvant. Statistical tests employed included the Mantel-Cox log-rank test, ANOVA and unpaired t-tests depending on the data analysed, with a post hoc Bonferroni adjustment for multiple measures.

**Results:**

Chimaeric RHDV VLP were found to form a composite particle in the presence of CpGs. Overall survival was significantly improved amongst mice bearing MC38-OVA tumours following vaccination with T.VP60 (60%, 9/15), S.VP60 (60%, 9/15) or TS.VP60 (73%, 11/15). TS.VP60 significantly prolonged the vaccine-induced remission period in comparison to each mono-therapy.

**Conclusions:**

Chimaeric VLP containing multiple epitopes were found to confer an advantage for therapeutic vaccination in a model of colorectal cancer based on the prolongation of remission prior to tumour escape.

**Electronic supplementary material:**

The online version of this article (doi:10.1186/s40425-017-0270-1) contains supplementary material, which is available to authorized users.

## Background

Colorectal cancer (CRC) represents a significant disease burden worldwide, with an incidence rate estimated to exceed 1.36 million cases annually [1]. Rates of CRC are particularly pronounced in New Zealand and Australia, with incidence rates more than double the world average of 20.6 and 14.3 per 100,000 people in men and women respectively, and high rates of metastatic disease on presentation [2]. Therapeutic options for treatment of CRC remain based on combinations of surgical resection, radiotherapy for rectal cancer, and chemotherapy; however, similar to other types of cancer, immunotherapy is becoming an increasingly popular therapeutic option for CRC. There remains a requirement for new therapeutic options for CRC possessing a combination of targeted, persistent and systemic efficacy, capable of combating distant metastases and preventing recurrence, while limiting off-target effects. Immunotherapy fits this model by targeting tumour-associated antigens (TAAs), overexpressed or selectively expressed by tumour cells.

Many TAAs are in a tolerised state with respect to the immune system amongst patients, requiring potent stimulation to disrupt tolerance. Therapeutic vaccine approaches for CRC can overcome this through the use of viral vectors, such as adenovirus [3], alphavirus [4], and vaccinia [5]; however, each of these vaccines possess specific design flaws that may impair their overall efficacy. These therapies target a single antigen, such as carcinoembryonic antigen (CEA), a TAA normally found expressed at low levels throughout the intestinal mucosa [6] and overexpressed in the majority of adenocarcinomas originating in either the colon or rectum [7]. Although CEA is a known target for therapeutic vaccination in CRC, expression of CEA may not be consistent throughout the heterogeneous tumour mass, and expression can be reduced following targeted vaccination [8, 9].

The closest murine analogues to well-known CRC-associated TAAs, such as CEA and mucin 1, possess differing expression profiles in mice that may impair pre-clinical vaccine validation [10, 11]. Topoisomerase IIα (topIIα) and survivin were selected as targets in this study, representing two independent survival mechanisms utilised by tumours with relevance in both murine models and humans. TopIIα is involved in chromosome segregation and replication, altering superhelices and the high order structural state of DNA, as well as disentangling interlinked chromosomes [12–14]. TopIIα expression is elevated in proliferating tissues [15], and is associated with various solid tumours including CRC [16]. In comparison, differentiated tissues do not normally express survivin [17]. Survivin plays an important role during angiogenesis, inhibiting apoptosis through mitochondria-dependent caspases [18], and is overexpressed in various tumour types [19]. Natural non-mutated epitopes from both topIIα and survivin have previously been investigated as targets for therapeutic CRC vaccines in mice [20, 21], and survivin has been investigated as a vaccine target in humans [22, 23]. However, these antigens have not been investigated for their synergistic potential in the form of a multi-target vaccine.

A multi-target vaccine covering a broader range of antigens expressed throughout the heterogeneous tumour, should promote tumour resolution by impairing the ability of the tumour to escape. The use of multi-target vaccines for cancer has generated mixed results, with immune responses dependent upon specific combinations of target antigens [24], providing both MHC-I and MHC-II-specific epitopes [25], and delivery of the vaccine aboard an immunogenic vehicle [26]. While each of these requirements may be covered by using a virus-based vaccine vector, the use of live viruses in vaccines does pose some safety concerns. Additionally, live virus vaccines are particularly prone to interference from pre-existing or induced anti-vector immunity.

An alternative approach that also meets the aforementioned criteria is the use of virus-like particles (VLP) as a delivery vector. VLP consist of non-infectious, non-replicative particles formed from self-polymerising virus capsid proteins, providing the appearance of a virus-based vaccine, with the safety profile of a subunit vaccine. Another advantage over the use of a live vaccine is that VLP are amenable to a broad range of modifications, including chemical conjugation, recombinant insertion, association with immunogenic molecules, and encapsulation of synthetic genetic material. As they are not reliant on infection to gain entry into host cells, some VLP vaccines are also unaffected by anti-vector immunity [27]. Rabbit haemorrhagic disease virus (RHDV) VLP have demonstrated particular promise as a potential vector for cancer vaccines [28–30], capable of cross-presenting immune epitopes to induce a cytotoxic immune response [31]. In this study, we have developed chimaeric RHDV VLP containing epitopes derived from CRC TAAs topIIα and survivin as an immunotherapeutic vaccine in a murine model of CRC, investigating the potential for therapeutic vaccination with a multi-target VLP vaccine.

## Methods

### Development of the T.VP60, S.VP60 and TS.VP60 constructs

Chimaeric RHDV VLP were developed using a recombinant baculovirus expression system, as has been previously published [32, 33]. In brief, the VP60 N-terminus for each construct was purchased as synthetic DNA in a pUC57-Simple plasmid (Genscript, New Jersey, USA). The N-terminus sequences were extracted and ligated with the remainder of RHDV VP60 in a pAcUW51 (GUS) expression plasmid. Expression plasmids were co-transfected into *Spodoptera frugiperda* (Sf21) cells grown in SF900-III medium (Invitrogen, Auckland, NZ) along with the FlashBAC ULTRA™ expression system (Oxford Expression Systems, Oxford, UK). Sequence identity was confirmed by sequencing (Massey Genome Service, NZ). Each recombinant baculovirus was plaque purified and amplified. VLP were expressed in Sf21 cells as has been previously published [33], including CsCl gradient purification by ultracentrifugation. Expressed recombinant VP60 constructs were confirmed through mass spectrometry using MALDI-TOF/TOF or a LTQ-Orbitrap hybrid (Centre for Protein Research, University of Otago, NZ).

### Detection of RHDV VLP

VLP expression was detected with separation by sodium dodecyl sulphate (SDS)-polyacrylamide gel electrophoresis (SDS-PAGE), and transferred onto nitrocellulose membrane using a Transblot SD blotter (Bio-Rad, California, USA). Membranes were probed by western blot using rabbit anti-VP60 (University of Otago, NZ) and DyLight 800-labelled donkey anti-rabbit monoclonal antibody (Clone SA5–10044, Lot QC1998302, Thermo Scientific, Delaware, USA), imaged using an Odyssey FC (Licor, Nebraska, USA). VLP assembly was confirmed by negative staining with phosphotungstic acid at pH 6.8 on carbon-coated grids, imaged on a CM100 BioTWIN transmission electron microscope (Philips/FEI Corporation, Eindhoven, Holland).

### In vivo cytotoxicity assay

Female C57BL/6 mice aged 6–8 weeks were assigned to each treatment in groups of 6 per assay. Vaccines were administered subcutaneously into the left flank on days 0 and 21, consisting of coupling PBS (CPBS) (0.03 M NaH_2_PO_4_.2H_2_O, 0.17 M Na_2_HPO_4_ and 0.15 M NaCl, pH 7.3), 100 μg VLP in CPBS, or equimolar quantities of synthetic non-mutant topIIα (DSDEDFSGL) and survivin (TVSEFLKL) peptides (JPT Peptide Technologies, Germany) in CPBS, each with 25 μg of ODN1826 CpGs (GeneWorks, Australia). Target cells were prepared from naïve C57BL/6 mouse splenocytes. Spleens were strained, washed, and red blood cells (RBCs) were lysed with ammonium chloride. Splenocytes were cultured in Iscove’s modified Dulbecco’s medium (IMDM) + 0.1% β-mercaptoethanol (cIMDM) (Gibco Invitrogen, New York, USA). Each target cell population was pulsed with 10 μM of assigned peptides for 2 h at 37 °C + 5% CO_2_, washed, and stained with one of four corresponding dye combinations: 0.2 μM carboxyfluorescein succinimidyl ester (CFSE^Lo^), 2 μM CFSE (CFSE^Hi^); 5 μM violet proliferation dye (VPD); and 2 μM CFSE + 5 μM VPD (CFSE^Hi^/VPD). Cells were stained for 7 min and quenched with FCS. Target cells were combined in equal proportions, and infused intravenously into vaccinated mice on day 28. Mice were culled on day 30, and splenocytes were prepared. Splenocytes were stained with near-infra-red (IR) live/dead stain (Life Technologies, California, USA) and fixed in 2% paraformaldehyde (PFA). Target populations were detected on a Gallios flow cytometer (Beckman Coulter, California, USA), and analysed using Kaluza version 1.2 (Beckman Coulter, California, USA). The gating strategy is provided in Additional file 1: Figure S1. Specific Lysis (%) was calculated using the equation below.

$$ \mathrm{Specific}\kern0.3em \mathrm{Lysis}\kern0.3em \left(\%\right)=\left[\kern0.3em \mathbf{1}-\left(\mathrm{Vaccine}:\mathrm{Target}\kern0.3em \mathrm{Cell}\#/\mathrm{Control}\kern0.3em \mathrm{Cell}\#\right)/\left(\mathrm{CPBS}:\mathrm{Target}\kern0.3em \mathrm{Cell}\#/\mathrm{Control}\kern0.3em \mathrm{Cell}\#\right)\kern0.3em \right]\times \mathbf{100} $$

### CpGs dialysis and analysis

Dialysis of CpGs with or without VLP was performed using 1 MDa dialysis tubing in CPBS for 24 h at 4 °C. Detection of CpGs was performed using 20% PAGE gels containing Tris/Borate/EDTA (TBE) buffer, labelled with Gel Green (Biotium, California, USA), imaged using an Odyssey FC.

### RAW-blue cell activation assay

RAW-blue (RB) cells (ATCC, Virginia, USA) were cultured in Dulbecco’s modified eagle medium (DMEM) GlutaMax (Gibco Invitrogen, New York, USA) containing 10% fetal bovine serum (FBS), 1X penicillin/streptomycin (Roche Diagnostics, Germany), 1X normocin (InvivoGen, California, USA) and 1X zeocin (Invitrogen, New York, USA) (RB-DMEM). RB cells were cultured overnight at 37 °C + 5% CO_2_ prior to treatment, with treatments including CPBS, 25 μg CpGs in CPBS pre- or post-dialysis, and 1 mg/mL VP60 VLP with or without CpGs pre- or post-dialysis. Treated cells were incubated for 24 h at 37 °C + 5% CO_2_, and media was harvested for secreted alkaline phosphatase (SEAP) quantification. SEAP was quantified using the QUANTI-Blue assay (InvivoGen, California, USA) as per the manufacturer’s instructions. Measurements were taken at 651 nm using a Multiskan microplate reader (MTX Lab Systems, Florida, USA), and concentrations of SEAP were determined relative to a concentration curve established from a 2-fold serial dilution of recombinant SEAP (InvivoGen, California, USA).

### BMDC activation assay

Bone marrow-derived dendritic cells (BMDCs) were derived from bone marrow washed from the rear limbs of C57BL/6 mice. Bone marrow cells were strained, washed, and RBCs lysed. The remaining cells were cultured in cIMDM +5% FCS with 20 ng/mL recombinant murine granulocyte macrophage colony stimulating factor (mGM-CSF) (ProSpec, New Jersey, USA), and incubated for 6 days at 37 °C + 5% CO_2_. BMDCs were harvested on day 6, plated at 1 × 10^6^ cells/mL in 200 μL cIMDM +5% FCS per well of a round-bottom 96-well plate, and treated with 10 μL of assigned treatments. Treatments included CPBS, CPBS with 10 μg CpGs pre- or post-dialysis, 1 μg lipopolysaccharide (LPS), and 1 mg/mL VP60 VLP with or without CpGs pre- or post-dialysis. Plates were incubated for 24 h at 37 °C + 5% CO_2_, and harvested for staining. Harvested BMDCs were stained with near-IR live/dead stain, labelled with anti-CD11c/APC (Clone N418), anti-CD40/PE-Cy7 (Clone 3123), anti-CD80/Pacific Blue (Clone 16-10A1), anti-CD86/PE (Clone GL-1) and anti-I-A/I-E/FITC (Clone M5/114.15.2) (BioLegend, California, USA), and fixed in 2% PFA. FACS analysis was performed on a Gallios flow cytometer, with data analysed using Kaluza version 1.2. The gating strategy is provided as Additional file 2: Figure S2.

### In vitro cytotoxicity assay

The in vitro cytotoxicity assay used was derived from the VITAL assay, originally described by Hermans et al. [34]. Female C57BL/6 mice aged 6–8 weeks were assigned to each treatment in groups of 9 per assay. Vaccines were administered subcutaneously into the left flank on days 0 (1D), 0 and 1 (2D) or 0, 1 and 2 (3D), consisting of 100 μg VLP and 25 μg CpGs in CPBS, 3 mice per vaccination regimen. Lymph nodes were resected on day 7, strained, washed and RBCs were lysed. CD8^+^ cells were sorted using CD8α (Ly-2) microbeads (Miltenyi Biotec, Germany) on an AutoMACS Pro cell separator (Miltenyi Biotec, Germany) by positive selection. Target cells were prepared as previously described, with the exception that a population pulsed with synthetic SIINFEKL peptide (JPT Peptide Technologies, Germany) was used. Sorted CD8^+^ T cells were co-cultured with target cells at an effector:target ratio of 0.1:1 for 4 h at 37 °C + 5% CO_2_. Cells were stained with near-IR live/dead stain and fixed with 2% PFA. Target populations were detected on a Gallios flow cytometer, and analysed using Kaluza version 1.2. The gating strategy is provided in Additional file 1: Figure S1.

### MC38-OVA tumour trials

MC38-OVA cells were provided by Dr. Nicole Haynes (Peter MacCallum Institute, Australia). Cells were cultured in DMEM +10% FCS at 37 °C + 5% CO_2_. 1 × 10^6^ MC38-OVA cells were injected subcutaneously into the left flank of female C57BL/6 mice aged 6–8 weeks. On day 7, tumour-bearing mice were assigned to treatment groups, balancing tumour range and size between groups. Vaccines were administered subcutaneously on the left flank on days 7, 8 and 9, consisting of CPBS +25 μg CpGs, or 1 mg/mL VLP + 25 μg CpGs. Tumours were measured every 2–3 days, and mice were culled when tumours reached 150 mm^2^ in cross-sectional area. On day 100, a group of naïve female C57BL/6 mice and the surviving mice from each treatment group received a rechallenge of 1 × 10^6^ MC38-OVA cells administered subcutaneously into the right flank. Mice were culled upon completion of the trial on day 200.

### Statistical analysis

Statistical analyses were calculated in Prism 6 (GraphPad, California, USA). An unpaired t-test was used when comparing two groups, otherwise 2-way ANOVA was used for multiple groups. Survival curve analysis was performed using the Mantel-Cox log-rank test. Accumulated error from multiple measurements was corrected with a post hoc Bonferroni adjustment.

## Results

### Production of chimaeric RHDV VLP

**Fig. 1:**
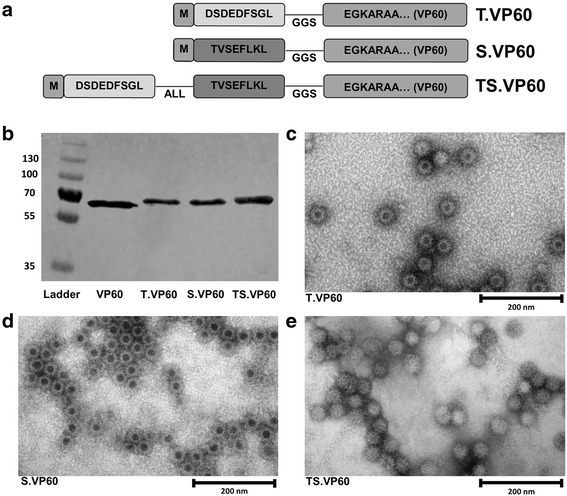
Chimaeric RHDV VLP Containing CRC TAAs. Each chimaeric RHDV VLP construct was expressed in Sf21 cells and purified for analysis and applications in vaccination. **a** Diagram outlining the structure of the recombinant VP60 produced for each chimaeric VLP construct, including non-mutant H-2K^b^ restricted peptide sequences, ALL linker and GGS spacer sequence. **b** Western blot probing for RHDV VP60 with T.VP60, S.VP60 and TS.VP60 represented in comparison to unmodified VP60. Electron microscopy was used to identify the formation of intact VLP from the expression of **c** T.VP60, **d** S.VP60 and **e** TS.VP60

Recombinant variants of RHDV VP60 capsid protein were developed by ligating synthetic N-terminus DNA sequences with the remainder of the *VP60* gene (Fig. 1a). Epitopes for topIIα and survivin were selected for recombinant insertion into VP60 based on published reports [20, 21]. The expression of each recombinant VP60 was detected by western blot for the RHDV VP60 major capsid protein (Fig. 1b), and the identity of each construct was confirmed by sequencing of the expression plasmid (Additional file 3: Figure S3A-C), and through mass spectrometry of purified VLP (Additional file 3: Figure S3D). RHDV VLP possess a *T* = 3 icosahedral structure, with protruding domains characteristic of *Caliciviridae* morphology [35]. This appearance is sometimes referred to as cog-like, and can be clearly identified by electron microscopy of RHDV VLP [36]. The chimaeric VLP constructs T.VP60, S.VP60 and TS.VP60 share this characteristic appearance when viewed by electron microscopy (Fig. 1c-e). Recombinant insertion of these epitopes did not appear to have a negative consequence upon the formation of intact VLP. The absence of dark central staining with phosphotungstic acid in TS.VP60 may be attributable to an increase in intraparticulate density from the insertion of a longer peptide.

### TopIIα and/or survivin chimaeric VLP induce target-specific cytotoxic responses

**Fig. 2:**
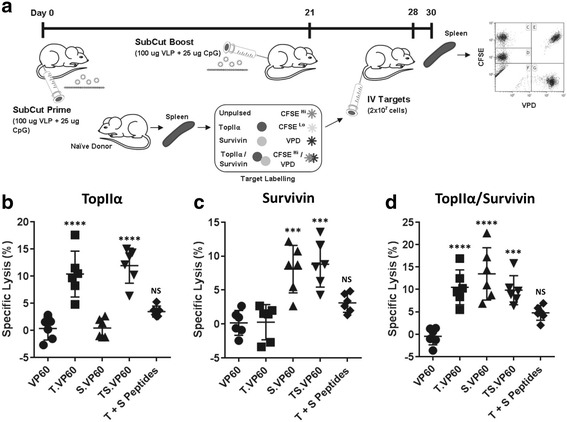
Induction of Target-specific Immunity with Chimaeric VLP. The ability of each chimaeric VLP construct to induce an immune response specifically targeting their incorporated epitope was assessed using a modified in vivo cytotoxicity assay. **a** Modified in vivo cytotoxicity protocol, using a combination of CFSE and VPD staining to simultaneously monitor cytotoxicity against multiple target populations. Responses were assessed for T.VP60, S.VP60 and TS.VP60 in comparison to unmodified VP60 or synthetic peptides, calculating specific lysis of the **b** topIIα, **c** survivin and **d** topIIα/survivin target populations. Results are representative of three independent repeats. Statistical analysis performed using unpaired t-tests. NS = Non-significant, *** *p* < 0.001, **** *p* < 0.0001

Chimaeric VLP constructs were assessed for their ability to induce target-specific cytotoxic immune responses using an in vivo cytotoxicity assay. Peptide/fluorophore labelled target cells were detected amongst splenocytes isolated from vaccinated mice 48 h post-intravenous infusion, 30 and 9 days following primary and boost vaccination respectively (Fig. 2a). Synthetic CpG DNA oligonucleotides (CpGs) were used as an adjuvant along with each vaccine. Splenocytes were gated to exclude doublets between side scatter area/height, gated for live cells, and for CFSE/VPD staining (Additional file 1: Figure S1A-C). Specific lysis (%) was calculated based on a comparison between target and non-target populations, with respect to the original proportions of these populations amongst the infused cells. When calculated with respect to the target population labelled with a combination of topIIα peptide/CFSE^Lo^, both T.VP60 and TS.VP60 induced responses capable of target-specific cytotoxicity of around 10% (Fig. 2b). Similarly, both S.VP60 and TS.VP60 induced responses capable of target-specific cytotoxicity with respect to the survivin peptide/VPD labelled population of around 5–10% (Fig. 2c). T.VP60, S.VP60 and TS.VP60 also induced responses capable of target-specific cytotoxicity with respect to the topIIα/survivin petide/CFSE^Hi^/VPD target population (Fig. 2d). The ability to induce these responses with both T.VP60 and S.VP60 against this dual peptide labelled population suggests that any preferential H-2k^b^ binding between these epitopes has had little to no effect. The inability of TS.VP60 to induce responses significantly increased over either T.VP60 or S.VP60 suggests that, regarding target-specific cytotoxicity, the simultaneous induction of responses against these two epitopes was not cumulative. The consistency of responses averaging around 10% may suggest that while it is possible to induce responses against these tolerised antigens, there are likely alternative mechanisms capable of dampening the efficacy of these responses in this setting. Mice vaccinated with the synthetic peptides did not generate significant responses.

### RHDV VLP naturally associate with CpG oligonucleotides

**Fig. 3:**
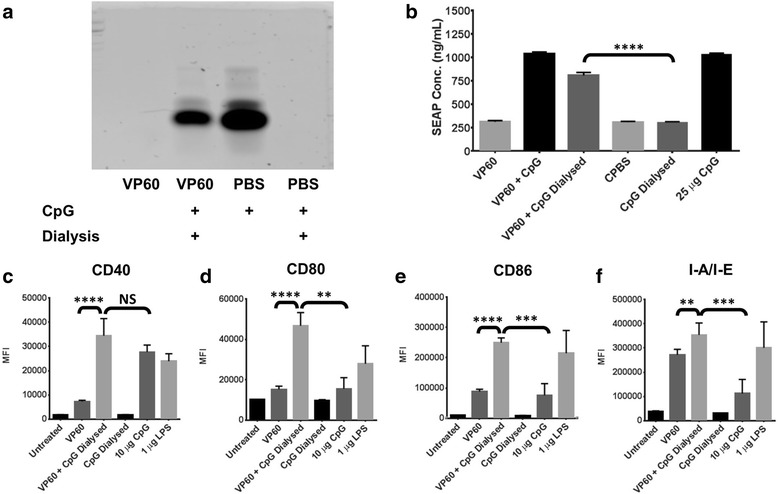
Association of CpG with RHDV VLP. The ability of RHDV VLP to naturally associate with CpG was investigated by dialysis with 1 MDa tubing. **a** CpGs were detected by TBE acrylamide gel electrophoresis stained with Gel Green following dialysis. **b** Quanti-blue assay on supernatants detecting SEAP secreted from the reporter cell line, RAW-blue, following treatment with CpG associated with VLP. This treatment was repeated with murine BMDCs, detecting the median fluorescence intensity (MFI) of the activation markers **c** CD40, **d** CD80, **e** CD86 and **f** I-A/I-E by flow cytometry. Results are representative of two independent repeats. L = Hyperladder V, Dial = Dialysed. Statistical analysis performed using unpaired t-tests. NS = Non-significant, ** *p* < 0.01, *** *p* < 0.001, **** *p* < 0.0001

CpGs have been investigated extensively as an adjuvant for VLP vaccines, previously used in combination with Qβ bacteriophage VLP [37], human papillomavirus VLP [38] and RHDV VLP [39]. In Qβ VLP, CpGs can be encapsulated into the intraparticulate space, facilitating co-delivery of the adjuvant and vaccine as a composite particle [37]. To investigate whether RHDV VLP forms as similar composite particle with CpGs, solutions containing VLP and CpGs were dialysed with 1 MDa tubing. When CpGs were dialysed alone with this tubing they were completely dialysed out of solution; however, in the presence of RHDV VLP they remained (Fig. 3a). This suggests that the presence of RHDV VLP in solution may provide some attractive force, impairing the ability of CpGs to defuse out of solution. The retention of CpGs was confirmed using the reporter cell line, RAW-blue. These cells are murine macrophages, modified to express secreted alkaline phosphatase (SEAP) in response to NF-κB activation, facilitating the detection of any immunostimulatory molecules present in solution. SEAP secretion following treatment of RAW-blue cells with the dialysed VLP and CpGs solution was significantly increased over a dialysed CpGs solution (Fig. 3b). The amount of CpGs associated with RHDV VP60 VLP following dialysis was calculated at around 20 μg CpGs per 1 mg of VLP (Additional file 4: Figure S4). The amount of CpGs is approximately doubled when associated with chimaeric RHDV VLP containing the L1 DNA-binding site of human papillomavirus type 16 (DBS.VP60), but was adversely affected by recombinant insertion of a positively charged octaarginine sequence (R8.VP60) (Additional file 4: Figure S4). The immunostimulatory capabilities of a composite particle formed from VLP and CpGs was assessed by treating naïve murine bone marrow-derived dendritic cells (BMDCs), gated by CD11c expression (Additional file 2: Figure S2). While the composite did not enhance surface expression of CD40 over CpGs delivered alone (Fig. 3c), CD80, CD86 and I-A/I-E were each significantly increased in surface expression (Fig. 3d-f). This indicates that the combination of VLP and CpGs delivered together is more immunogenic than when administered separately, possibly due to differences in the uptake and detection of CpGs as a composite particle with VLP.

### Cytotoxic responses induced by chimaeric VLP can be boosted with multiple vaccinations

**Fig. 4:**
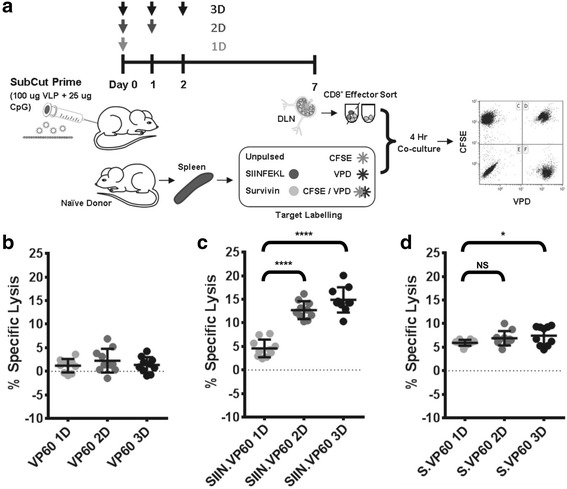
Comparison of Multiple VLP Vaccination Doses. Boosting of epitope-specific immune responses through multiple vaccination doses was investigated using a modified in vitro cytotoxicity assay. **a** Modified in vitro cytotoxicity assay, using a combination of CFSE and VPD to simultaneously monitor cytotoxicity against multiple target populations. **b** Cytotoxicity against survivin labelled target cells in mice vaccinated with VP60 on day 0 (1D), day 0 and 1 (2D) and days 0, 1 and 2 (3D). Results were comparable when calculated with respect to the SIINFEKL labelled population. Cytotoxicity was compared between mice vaccinated 1D, 2D or 3D with **c** SIIN.VP60 or **d** S.VP60 for their respective target populations to investigate whether an advantage in immunogenicity was gained through multiple dosages. Results are representative of three independent repeats. Statistical analysis performed using unpaired t-tests. NS = Non-significant, * *p* < 0.05, **** *p* < 0.0001

The infection cycle of RNA viruses, such as those of the *Caliciviridae* family to which RHDV belongs, tends to reach peak virus titre at around 72 h post infection [40, 41]. While RHDV VLP vaccines have traditionally been administered as a single dose vaccine, it was thought that mimicking the infection cycle through repeated administration over a 3-day period may induce immune responses with increased potency, despite being well short of the normal suitable window for boosting cytotoxic immune responses. This concept was investigated using an in vitro cytotoxicity assay, co-culturing sorted CD8^+^ T cells with peptide/fluorophore labelled target cells for 4 h, 7 days after initial vaccination. Vaccines were administered on day 0 (1D), days 0 and 1 (2D) or days 0, 1 and 2 (3D) (Fig. 4a). CpGs were used as an adjuvant. Co-cultures were analysed by flow cytometry, gating to exclude doublets and dead cells, and gated for CFSE/VPD staining (Additional file 1: Figure S1D-F). The specific lysis (%) was calculated for groups administered with VP60 VLP using each vaccine regime against both the survivin peptide/CFSE (Fig. 4b) and SIINFEKL/VPD labelled populations, generating similar negative results. SIIN.VP60 administered with either 2D or 3D treatment resulting in a significant increase in target-specific responses in comparison to 1D, but there was no significant difference between 2D and 3D treatment (Fig. 4c). When translated to the survivin epitope with S.VP60, only the 3D regime was significantly increased over 1D (Fig. 4d). This increase was small compared to those achieved with the SIINFEKL peptide, suggesting that the benefit gained through multi-dose vaccination against tolerised epitopes may be impaired; however, small differences in responses induced during initial vaccination may have a larger impact in subsequent applications.

### TopIIα and/or survivin chimaeric VLP delay tumour growth and enhance survival amongst MC38-OVA tumour-bearing mice

**Fig. 5:**
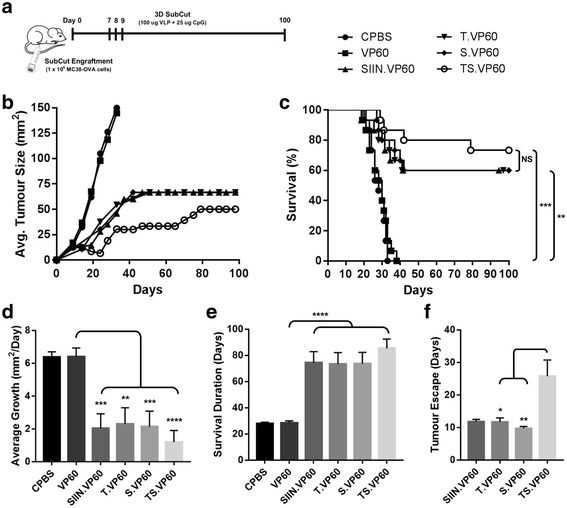
MC38-OVA Tumour Trial with Immunotherapeutic Vaccination. The capability of chimaeric RHDV VLP to induce immune responses against subcutaneously engrafted MC38-OVA tumours was investigated with tumour trials. **a** Tumour engraftment and vaccination protocol, with MC38-OVA tumours engrafted subcutaneously on Day 0, vaccination on days 7, 8 and 9, and rechallenge with MC38-OVA cells in the opposing flank on day 100. **b** Tumour growth rate and **c** overall survival following primary challenge of mice vaccinated with T.VP60, S.VP60 and TS.VP60 in comparison to CPBS, VP60 and SIIN.VP60. **d** The average growth rate of tumours was calculated based on the change in cross-sectional area (mm^2^) between each measurement divided by the number of days between those measurements. **e** Comparison of the survival duration amongst tumour-bearing mice between treatment groups. **f** Determination of tumour escape events, defined as the first positive growth rate measurement following vaccination-induced tumour regression. Results were combined from two independent repeats. Statistical analysis performed using Mantel-Cox log-rank tests for Kaplan-Meier survival curve. NS = Non-significant, * *p* < 0.05, ** *p* < 0.01, *** *p* < 0.001, **** *p* < 0.0001

The efficacy of each chimaeric VLP to induce therapeutic responses against established CRC tumours was investigated in C57BL/6 mice, using the murine CRC cell line MC38-OVA. Expression of topIIα and survivin in MC38-OVA cells was confirmed by western blot (Additional file 5: Figure S5). Tumours were established subcutaneously in the right flank of mice on day 0, with vaccination following the 3D regime on days 7, 8 and 9. Mice were followed for a period of 100 days (Fig. 5a). The mono-target vaccines T.VP60 and S.VP60 induced responses capable of delaying tumour growth, significantly improving survival to 60% (*N* = 9/15), levels comparable with the positive control SIIN.VP60 (Fig. 5b-c). The multi-target vaccine TS.VP60 further delayed tumour growth, approaching near universal remission around days 21-24, before tumours escaped in a proportion of the mice. These results translated into survival of 73% (*N* = 11/15), which although trending above the mono-target vaccines in each repeated trial, was not cumulatively statistically significant (Fig. 5b-c). These trends were also found to translate into reductions in average tumour growth rate (Fig. 5d), and the overall duration of survival between treatment groups (Fig. 5e). Interestingly, mice vaccinated with TS.VP60 demonstrated a significant delay in the observation of tumour escape following vaccination-induced remission (Fig. 5f). This can also be observed within the tumour growth curve, delaying the outgrowth in tumours amongst mice vaccinated with TS.VP60. Recurrent tumours developed following this delay in the TS.VP60 grew rapidly and aggressively upon emergence, reaching the assigned ethical cut-off within a shorter time period than most primary tumours (Additional file 6: Figure S6). MC38-OVA tumours were found to reach this cut-off after around 27.5 days on average, ranging between 19 and 35 days.

**Fig. 6:**
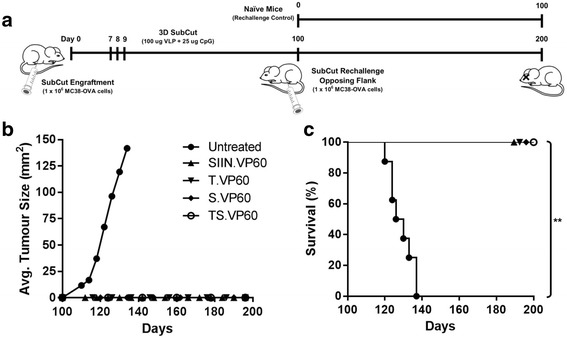
MC38-OVA Rechallenge in Opposing Flank. A rechallenge with a secondary tumour is used to evaluate the establishment of systemic anti-tumour immunity following vaccination-induced recovery from a primary tumour. **a** Mice that were tumour-free at day 100 of each MC38-OVA tumour trial were rechallenged with a second engraftment of MC38-OVA cells in the opposing flank. A group of naïve C57BL/6 mice was used as a control for engraftment viability. **b** Tumour growth rate and **c** overall survival following secondary challenge with subcutaneous MC38-OVA tumours. Results were combined from two independent repeats. Statistical analysis performed using Mantel-Cox log-rank tests for Kaplan-Meier survival curve. NS = Non-significant, * *p* < 0.05, ** *p* < 0.01, *** *p* < 0.001, **** *p* < 0.0001

Mice that survived their primary tumour challenge, achieved complete remission, and were tumour-free at day 100 were subsequently rechallenged with a second MC38-OVA tumour in the opposing flank (Fig. 6a). A naïve untreated population was used as a negative control, with mice from the vaccine groups SIIN.VP60 (*N* = 9), T.VP60 (*N* = 9), S.VP60 (*N* = 9) and TS.VP60 (*N* = 11) rechallenged. While the control population succumbed to their tumours within the expected period, none of the mice from each of the vaccine groups grew a rechallenge tumour, resulting in 100% survival through to completion of the trial at 200 days (Fig. 6b-c). The inability to establish tumours in the opposing flank of vaccinated mice suggests the presence of a systemic memory response sufficient to impair engraftment. The expression of ovalbumin by MC38-OVA potentially confounds the interpretation of the responses observed with these vaccines, and these experiments have been repeated using MC38. Preliminary results indicate that while overall survival dropped to 20% and 40% for the mono- and multi-target vaccines respectively, similar trends were observed in the non-OVA model for both average tumour growth and survival (Additional file 7: Figure S7A-C).

## Discussion

All currently approved immunotherapies for CRC consist of monoclonal antibodies (mAbs), and include the likes of the anti-vascular endothelial growth factor (VEGF) mAb bevacizumab (Genentech), and the anti-epidermal growth factor receptor (EGFR) mAbs cetaximab (ImClone LLC) and panitumumab (Amgen) [42–44]. Immune checkpoint inhibitors, such as the anti-cytotoxic T lymphocyte-associated protein 4 (CTLA-4) mAb ipilimumab (Bristol-Meyers Squibb) and the anti-programmed cell death protein 1 (PD1) mAbs nivolumab (Bristol-Meyers Squibb) and tremelimumab (Pfizer) remain in clinical trials for treatment of CRC (NCT02060188, NCT01975831, NCT02754856). There are currently no therapeutic vaccines for CRC approved for clinical administration, although several are currently in phase I trials [5, 45, 46]. The vaccines developed for each of these trials target a single TAA, reliant upon bystander responses induced during targeted elimination to compensate for vaccine-induced tumour evolution and escape.

Targeting a single highly expressed marker is not necessarily a limited design choice, such as when that target is a critical contributor to the development and sustainment of that tumour. Prominent examples of this include *HER2* mutant breast cancer, or *BCR-ABL1* positive chronic myelogenous leukaemia, both of which respond well to targeted therapies [47–49]. Many tumours instead may possess or acquire redundancies, oncogenic metabolic pathways capable of compensating for the loss or downregulation of a core component. Such redundancy may also be represented through tumour evolution, with the development of a polyclonal heterogeneous tumour mass. The concept of multi-target vaccination is to limit the ability of the tumour to escape, by targeting more of its potential redundancies. Some emerging vaccines share this vision for multi-target vaccination, including several in clinical trials for CRC [50–52].

When administered as a composite particle associated with CpGs, chimaeric VLP containing epitopes derived from these TAAs were capable of breaking tolerance, inducing target-specific immune responses. Importantly, these responses were induced simultaneously by the multi-target construct, indicating that immunodominance does not appear to play a role in this epitope combination. This is an important consideration in a multi-target vaccine as immunodominance can play roles at multiple levels through response induction, including during thymic selection of T cells [53], and competition between activated T cells during expansion [54]. Relative abundance and efficient processing are known to be important determinants for inducing simultaneous responses against multiple epitopes [55]. Delivery within chimaeric VLP holds a distinct advantage in this regard, with each particle containing 180 copies of each epitope delivered together within an immunogenic construct. The ability to form a composite particle in association with adjuvants such as CpGs and α-galactosylceramide [32] further enhances the versatility of RHDV VLP as a vaccine construct.

Targeting topIIα and survivin individually, or as a multi-target therapy proved effective as a therapeutic treatment in a murine model of CRC. The multi-target vaccine was at least as efficacious as each mono-target vaccine, with trends suggesting an improvement in both delaying tumour growth, and overall survival. The tumour growth kinetics following vaccination with TS.VP60, especially with the significantly delayed emergence of recurrent tumours, did indicate that targeting both topIIα and survivin simultaneously altered the capacity for tumours to evolve and escape. However, as some tumours were still able to escape following this therapy, additional evolutionary pathways and redundancies must remain. Increasing the number of TAAs targeted may further impede tumour escape, though this would require careful design to deal with the increased complexity of the vaccine and the potential for immunodominance. Those mice that achieved complete regression following vaccination with either the mono- or multi-target vaccines were completely protected from rechallenge with the same tumour in the opposing flank. This may represent the establishment of robust, targeted responses induced by these vaccines, or the induction of bystander responses against immunogenic molecules expressed by the MC38-OVA cells. Further investigation into the immunological mechanisms involved in multi-target vaccination may provide insight into these processes, leading to the development of a novel CRC vaccine suitable for translation into human clinical trials.

## Conclusions

Chimaeric RHDV VLP containing colorectal cancer TAAs are a viable candidate vaccine for translation from murine models to human clinical trials, especially when delivered as a composite particle with CpGs. The use of multiple epitopes in a chimaeric VLP construct did not have deleterious effects on individual immune responses, indicating that these can be induced simultaneously without obvious immunodominance. The primary benefit conferred by targeting multiple epitopes appears to be the prolongation of the vaccine-induced remission period prior to tumour escape. This may correspond with the equilibrium phase of the hypothesised immunoediting underlying therapeutic vaccination for cancer. The induction of multiple simultaneous immune responses targeting TAAs may apply additional evolutionary constraints upon tumours, prolonging the ability for tumours to develop resistance, or escape through the proliferation of a resistant subpopulation. The incorporation of additional epitopes should therefore exacerbate this effect, so long as the combination used is unaffected by immunodominance. It may thus be possible to identify a combination of epitopes capable of complete tumour eradication.

## Additional files

Additional file 1: Figure S1. In vivo and in vitro Cytotoxicity Gating Strategies. Gating strategy used for identification of target cell populations in an in vivo cytotoxicity assay. (a) Doublets were excluded by comparing side scatter (SS) interval (INT) against SS height (PEAK). (b) Live cells were gated as negative for Near-IR Live/Dead. (c) Target cell populations were identified by comparing CFSE and VPD staining, giving CFSE^Hi^, CFSE^Lo^, VPD and CFSE^Hi^/VPD stained populations. Unstained cells from Gate F were partially masked to improve the comparison of proportions between the stained target populations. A similar gating strategy was used for identification of target cell populations in an in vitro cytotoxicity assay. (d) Doublets were excluded by comparing SS INT against SS PEAK. (e) Live cells were gated as negative for Near-IR Live/Dead. (f) Target cell populations were identified by comparing CFSE and VPD staining, giving CFSE^Hi^, VPD and CFSE^Hi^/VPD stained populations. Unstained cells from Gate F were left unmasked. (JPEG 752 kb)

Additional file 2: Figure S2. BMDC Activation Assay Gating Strategy. Gating strategy used for determining the surface expression of BMDC activation markers. (a) Non-cellular debris was excluded in a comparison between forward scatter (FS) INT and SS INT. (b) Doublets were excluded by comparing SS INT against SS PEAK. (c) Live cells were gated as negative for Near-IR Live/Dead. (d) CD11c^+^ cells were selected by gating for CD11c/APC. Positive expression of each marker was identified with gates specific for detection of (e) CD40/PE-Cy7, (f) CD80/Pacific Blue, (g) CD86/PE and (h) I-A/I-E/FITC. Median Fluorescence Intensity (MFI) was determined from expression over the whole CD11c^+^ population. (JPEG 857 kb)

Additional file 3: Figure S3. Confirmation of Chimaeric RHDV VLP Constructs. The development of new chimaeric RHDV VLP constructs includes the identity confirmation using a combination of sequencing and mass spectrometry. The identity was initially confirmed by sequencing of the expression plasmid for (a) T.VP60, (b) S.VP60 and (c) TS.VP60. (d) Identity was further confirmed by analysing recombinant VP60 extracts using mass spectrometry, with underlined portions indicating peptide fragments identified by MALDI-TOF/TOF or a LTQ-Orbitrap hybrid. (JPEG 755 kb)

Additional file 4: Figure S4. Quantification of CpGs Associated with RHDV VLP. CpGs associated with RHDV VLP post-dialysis were detected and quantified using a combination of TBE acrylamide gel electrophoresis, staining with GelGreen dye (Biotium, California, USA), and determination of band intensity on an Odyssey FC. (a) CpGs associated with VP60 VLP was compared to two chimaeric VLPs containing recombinantly inserted regions with DNA-binding or associating properties (R8.VP60 and DBS.VP60). (b-c) A concentration curve was established for CpGs using the same method. (d) The amount of CpG present associated with each VLP was determined, with VP60 quantification performed by western blot analysed using an Odyssey FC. (JPEG 274 kb)

Additional file 5: Figure S5. Expression of TopIIα and Survivin. The expression of both topIIα and survivin in MC38-OVA cells was confirmed by western blot, and was compared against the expression of these proteins in MC38 cells. (JPEG 320 kb)

Additional file 6: Figure S6. Individual Tumour Growth Curves. The tumour growth curves for individual mice are provided from one representative tumour trial, with treatment groups including (a) CPBS, (b) VP60, (c) SIIN.VP60, (d) T.VP60, (e) S.VP60 and (f) TS.VP60. (JPEG 723 kb)

Additional file 7: Figure S7. MC38 Tumour Trial. Investigation of the chimaeric RHDV VLP against subcutaneously engrafted MC38 murine colorectal cancer tumours. (a) Tumour engraftment and vaccination protocol, with MC38 tumours engrafted subcutaneously on Day 0, vaccination on days 7, 8 and 9, and rechallenge with MC38 cells in the opposing flank on day 70. (b) Tumour growth rate and (c) overall survival following primary challenge and rechallenge of mice vaccinated with T.VP60, S.VP60 and TS.VP60 in comparison to CPBS and VP60. An age-matched naïve population of C57BL/6 mice was used as a rechallenge control group. Statistical analysis performed using Mantel-Cox log-rank tests for Kaplan-Meier survival curve. NS = Non-significant, * *p* < 0.05. (JPEG 291 kb)

## Abbreviations

BMDCs: Bone marrow-derived dendritic cells
CEA: Carcinoembryonic antigen
CpGs: Unmethylated CpG DNA Oligonucleotides
CRC: Colorectal cancer
CTLA-4: Cytotoxic T Lymphocyte Associated protein 4
EGFR: Epidermal growth factor receptor
PD1: Programmed Death 1
RHDV: Rabbit haemorrhagic disease virus
TAAs: Tumour-associated antigens
TopIIα: Topoisomerase IIα
VEGF: Vascular endothelial growth factor
VLP: Virus-like particle
