# Supplementary figures and images for: Multi-target chimaeric VLP as a therapeutic vaccine in a model of colorectal cancer
Source: J Immunother Cancer. 2017 Aug 15;5:69. doi: 10.1186/s40425-017-0270-1 (PMC5556368; doi:10.1186/s40425-017-0270-1)

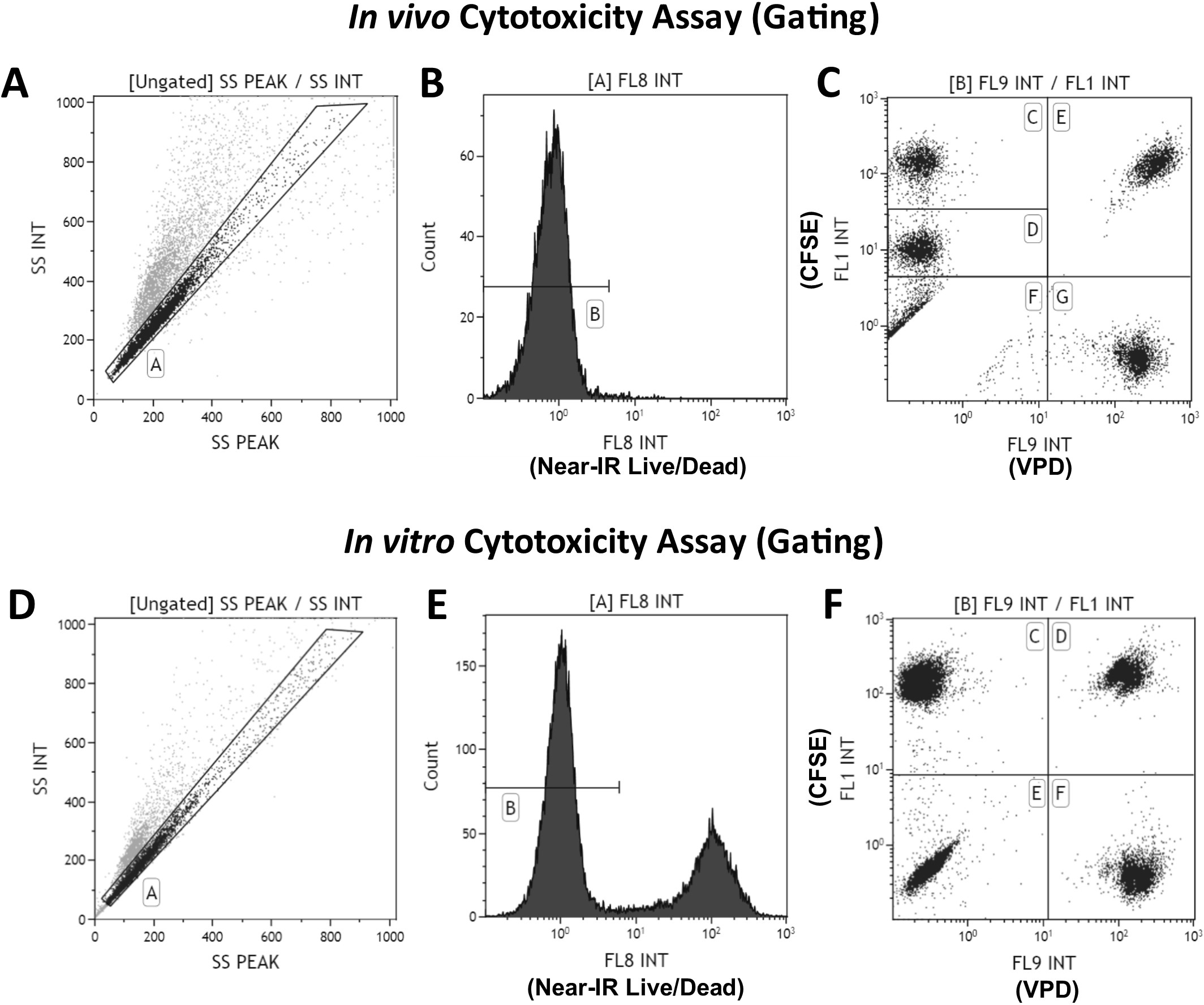

Supplement: Supplementary file 1 — In vivo and in vitro Cytotoxicity Gating Strategies. Gating strategy used for identification of target cell populations in an in vivo cytotoxicity assay. (a) Doublets were excluded by comparing side scatter (SS) interval (INT) against SS height (PEAK). (b) Live cells were gated as negative for Near-IR Live/Dead. (c) Target cell populations were identified by comparing CFSE and VPD staining, giving CFSEHi, CFSELo, VPD and CFSEHi/VPD stained populations. Unstained cells from Gate F were partially masked to improve the comparison of proportions between the stained target populations. A similar gating strategy was used for identification of target cell populations in an in vitro cytotoxicity assay. (d) Doublets were excluded by comparing SS INT against SS PEAK. (e) Live cells were gated as negative for Near-IR Live/Dead. (f) Target cell populations were identified by comparing CFSE and VPD staining, giving CFSEHi, VPD and CFSEHi/VPD stained populations. Unstained cells from Gate F were left unmasked. (JPEG 752 kb) [file 40425_2017_270_MOESM1_ESM.jpg]

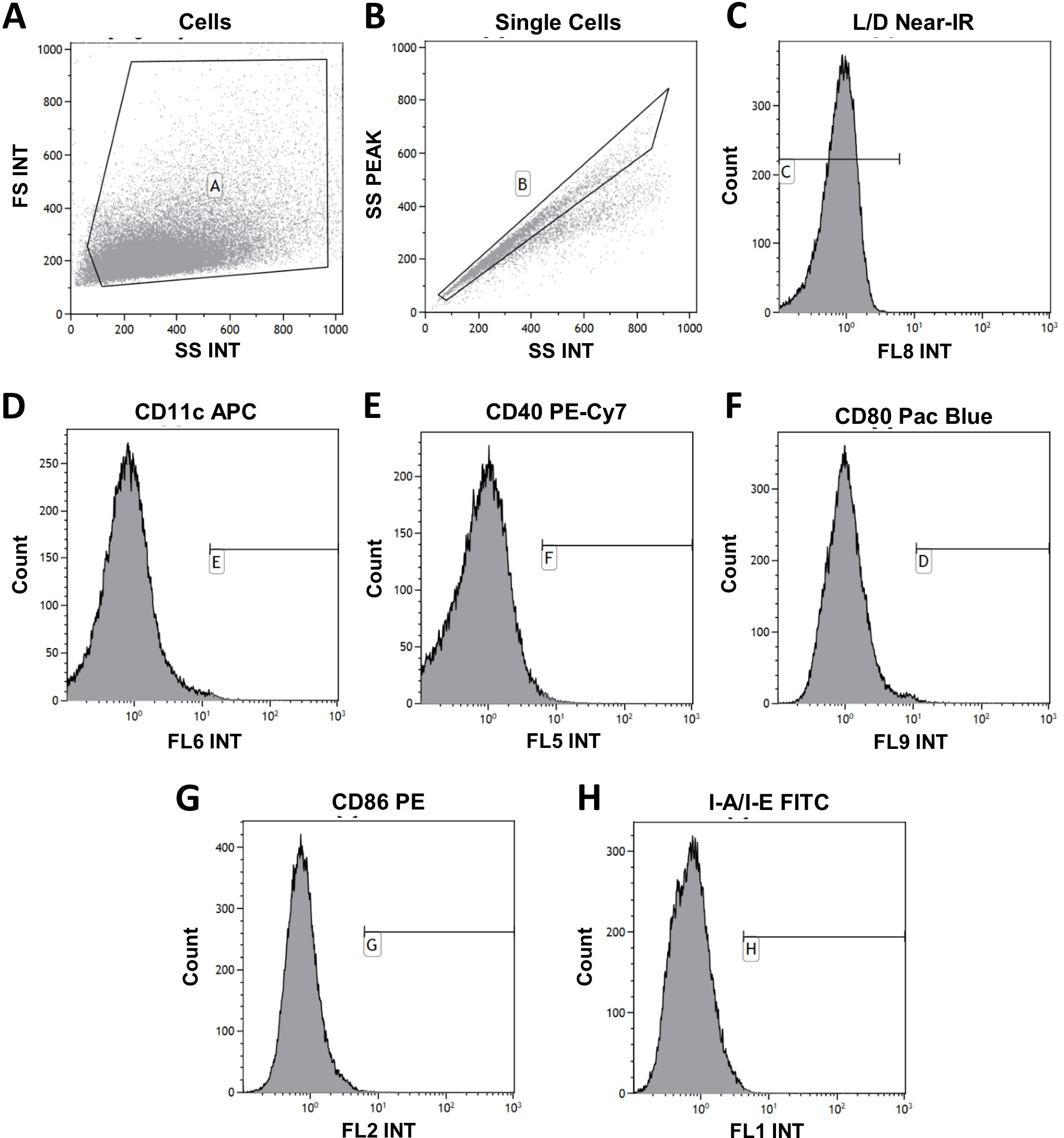

Supplement: Supplementary file 2 — BMDC Activation Assay Gating Strategy. Gating strategy used for determining the surface expression of BMDC activation markers. (a) Non-cellular debris was excluded in a comparison between forward scatter (FS) INT and SS INT. (b) Doublets were excluded by comparing SS INT against SS PEAK. (c) Live cells were gated as negative for Near-IR Live/Dead. (d) CD11c+ cells were selected by gating for CD11c/APC. Positive expression of each marker was identified with gates specific for detection of (e) CD40/PE-Cy7, (f) CD80/Pacific Blue, (g) CD86/PE and (h) I-A/I-E/FITC. Median Fluorescence Intensity (MFI) was determined from expression over the whole CD11c+ population. (JPEG 857 kb) [file 40425_2017_270_MOESM2_ESM.jpg]

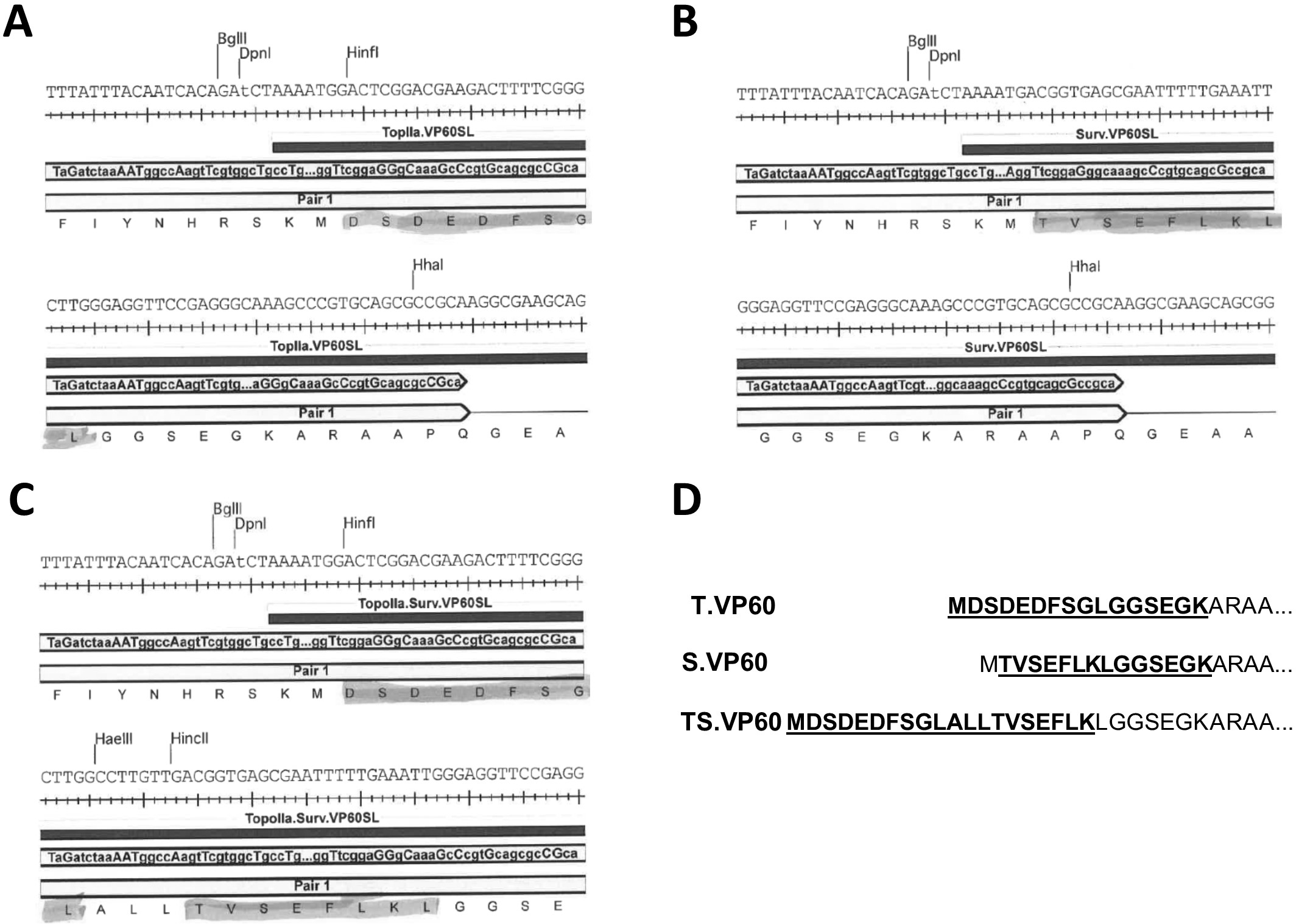

Supplement: Supplementary file 3 — Confirmation of Chimaeric RHDV VLP Constructs. The development of new chimaeric RHDV VLP constructs includes the identity confirmation using a combination of sequencing and mass spectrometry. The identity was initially confirmed by sequencing of the expression plasmid for (a) T.VP60, (b) S.VP60 and (c) TS.VP60. (d) Identity was further confirmed by analysing recombinant VP60 extracts using mass spectrometry, with underlined portions indicating peptide fragments identified by MALDI-TOF/TOF or a LTQ-Orbitrap hybrid. (JPEG 755 kb) [file 40425_2017_270_MOESM3_ESM.jpg]

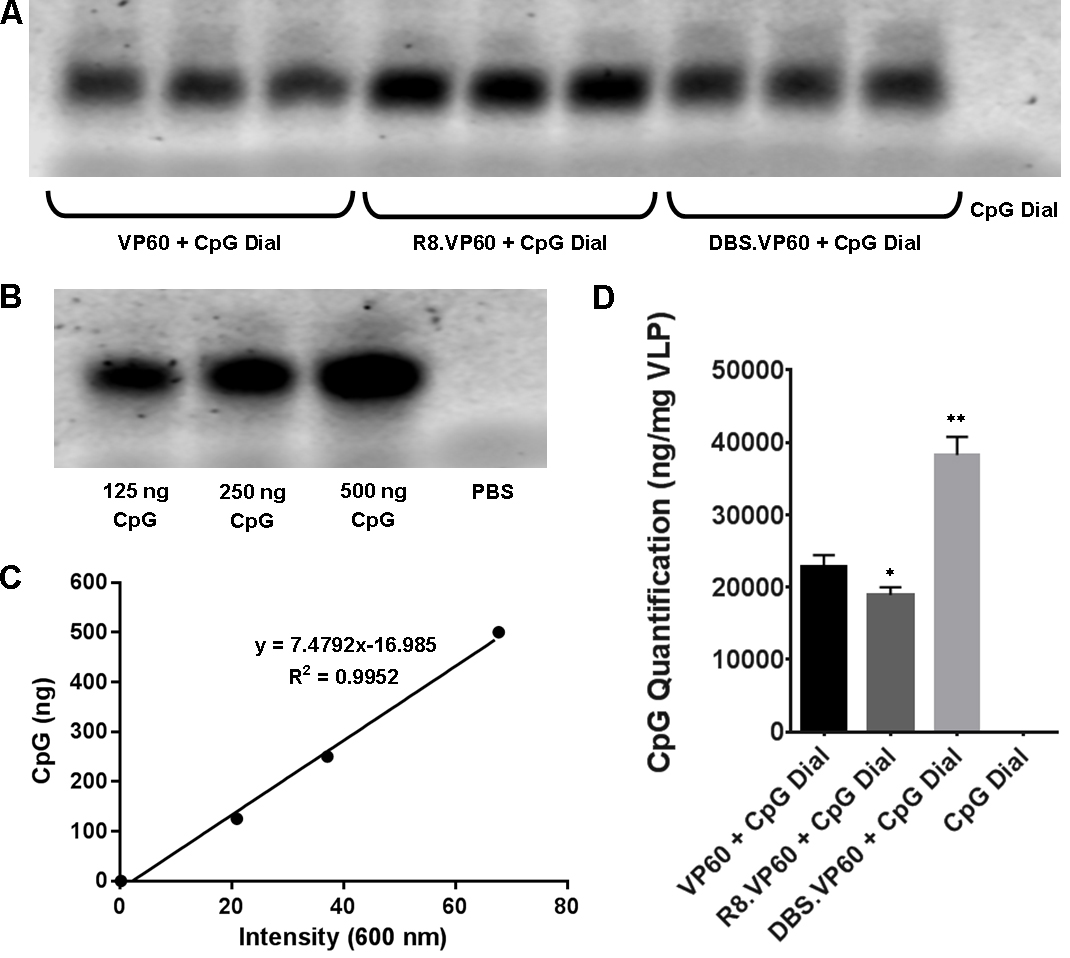

Supplement: Supplementary file 4 — Quantification of CpGs Associated with RHDV VLP. CpGs associated with RHDV VLP post-dialysis were detected and quantified using a combination of TBE acrylamide gel electrophoresis, staining with GelGreen dye (Biotium, California, USA), and determination of band intensity on an Odyssey FC. (a) CpGs associated with VP60 VLP was compared to two chimaeric VLPs containing recombinantly inserted regions with DNA-binding or associating properties (R8.VP60 and DBS.VP60). (b-c) A concentration curve was established for CpGs using the same method. (d) The amount of CpG present associated with each VLP was determined, with VP60 quantification performed by western blot analysed using an Odyssey FC. (JPEG 274 kb) [file 40425_2017_270_MOESM4_ESM.jpg]

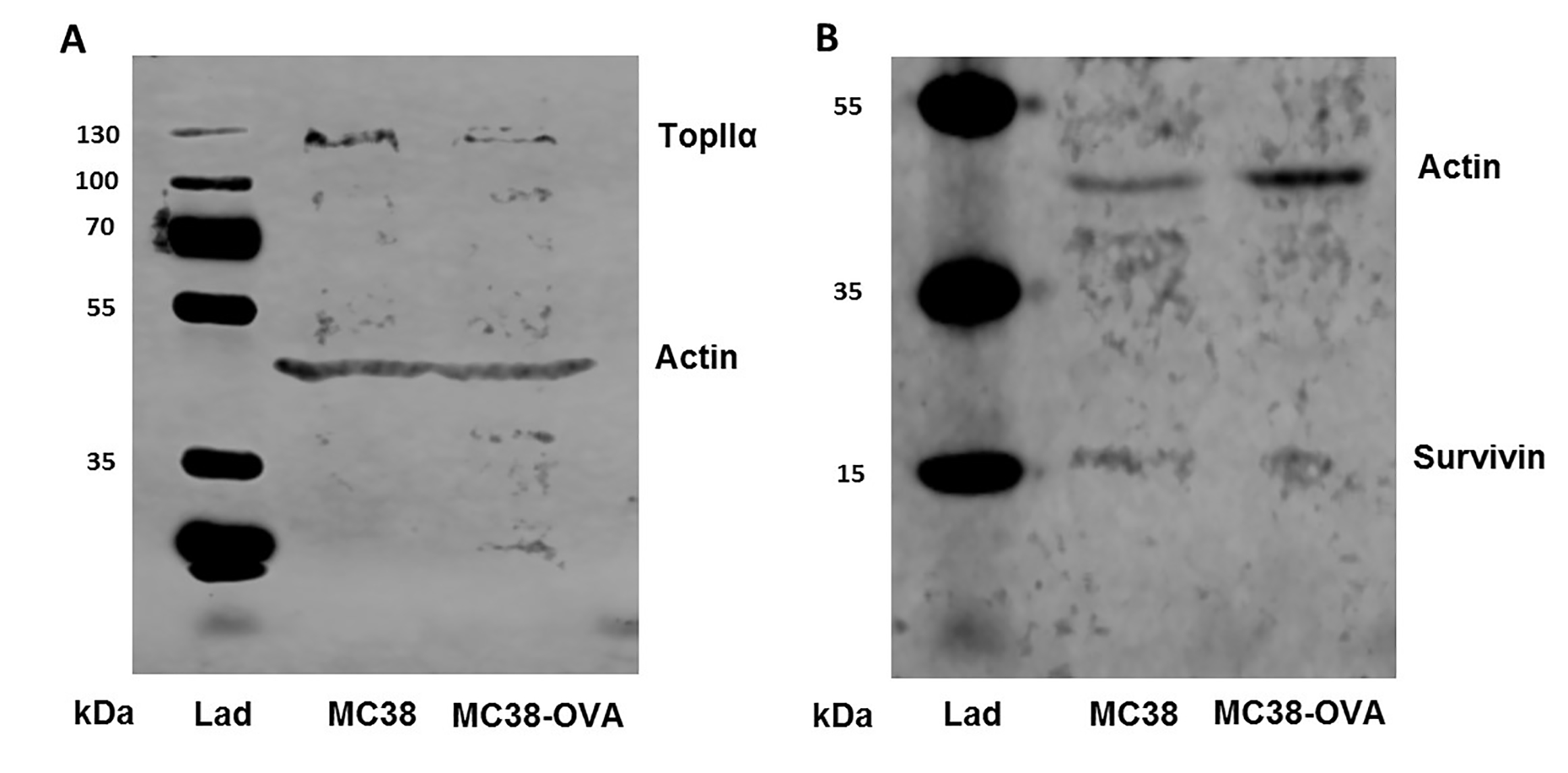

Supplement: Supplementary file 5 — Expression of TopIIα and Survivin. The expression of both topIIα and survivin in MC38-OVA cells was confirmed by western blot, and was compared against the expression of these proteins in MC38 cells. (JPEG 320 kb) [file 40425_2017_270_MOESM5_ESM.jpg]

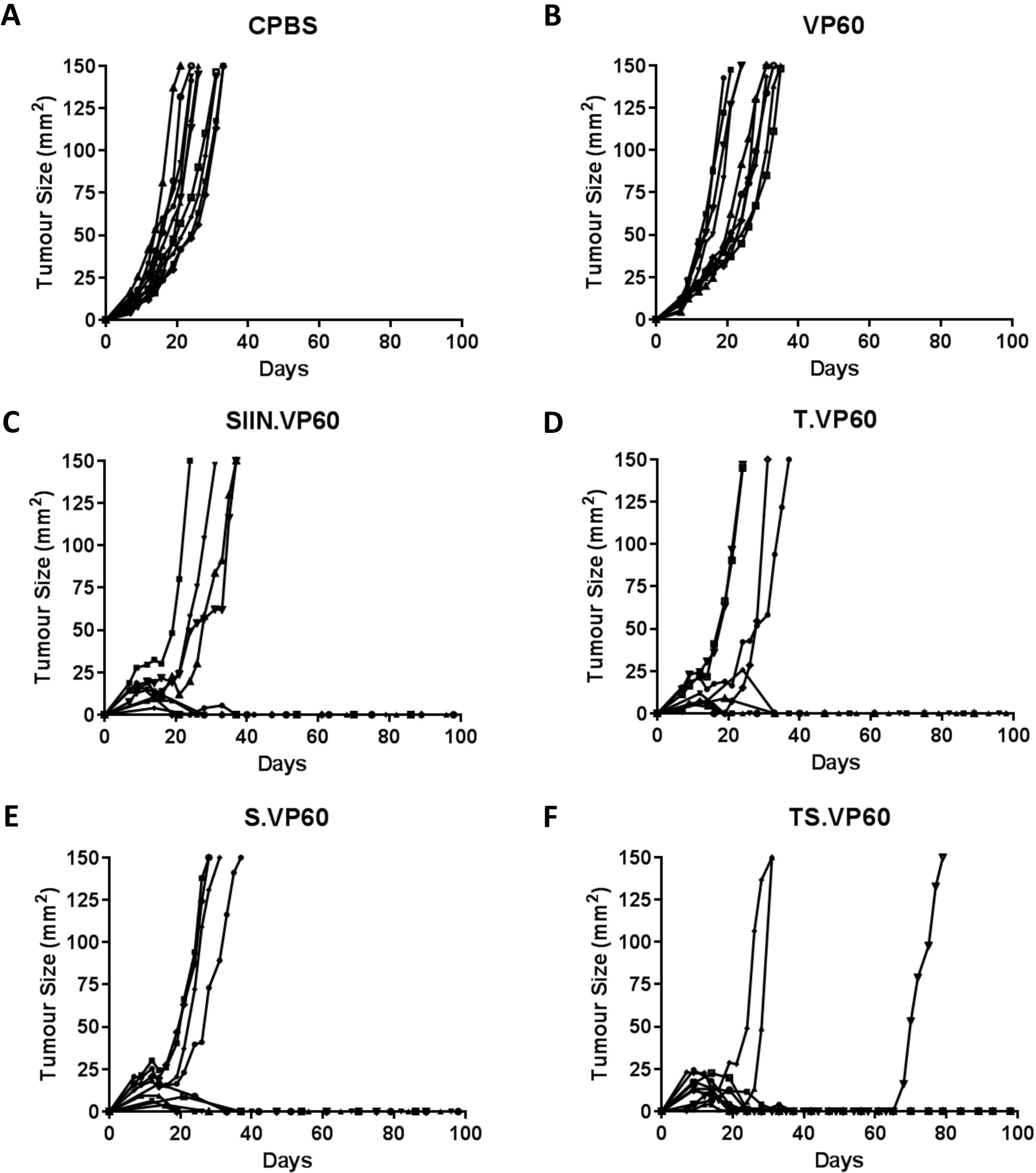

Supplement: Supplementary file 6 — Individual Tumour Growth Curves. The tumour growth curves for individual mice are provided from one representative tumour trial, with treatment groups including (a) CPBS, (b) VP60, (c) SIIN.VP60, (d) T.VP60, (e) S.VP60 and (f) TS.VP60. (JPEG 723 kb) [file 40425_2017_270_MOESM6_ESM.jpg]

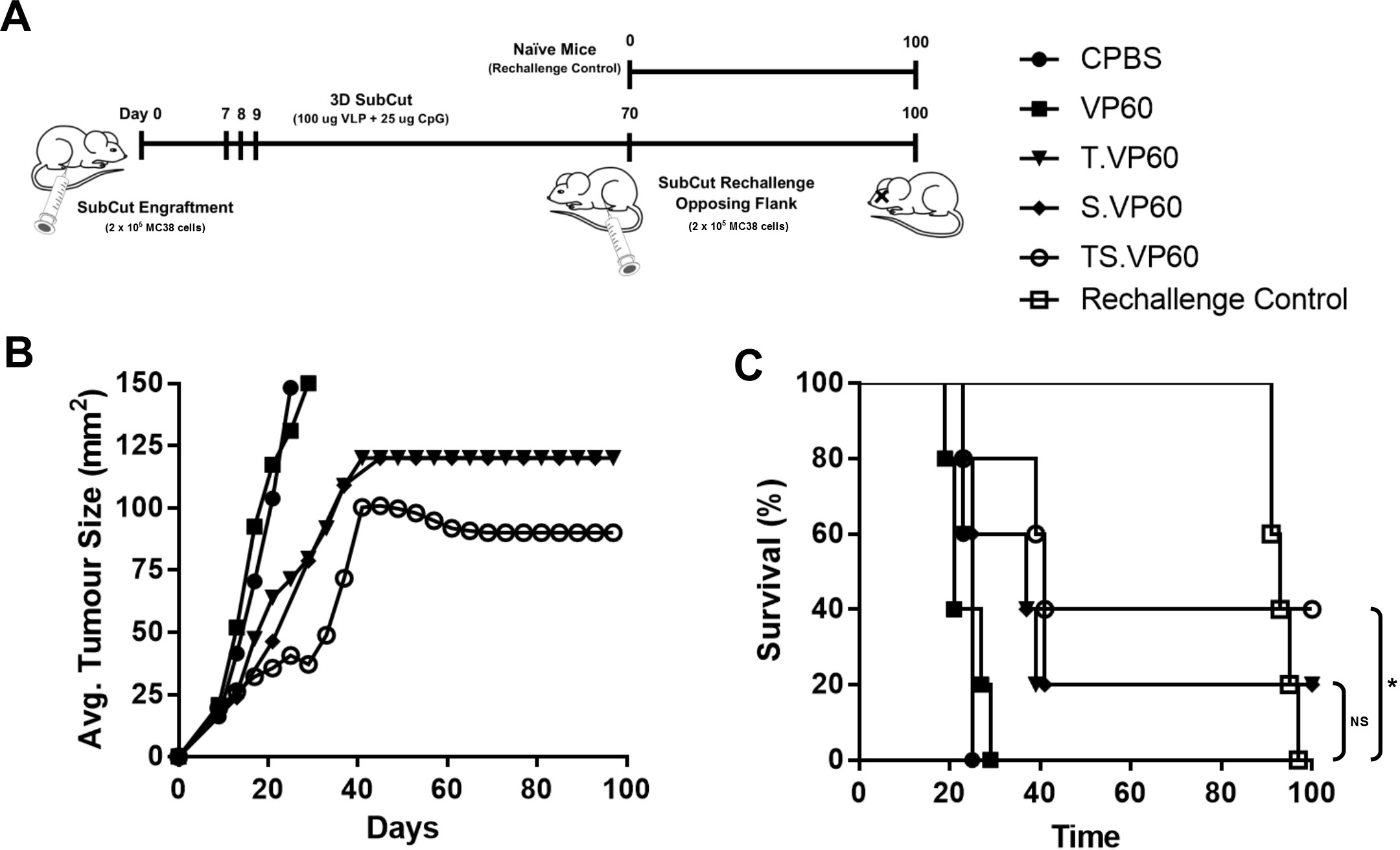

Supplement: Supplementary file 7 — MC38 Tumour Trial. Investigation of the chimaeric RHDV VLP against subcutaneously engrafted MC38 murine colorectal cancer tumours. (a) Tumour engraftment and vaccination protocol, with MC38 tumours engrafted subcutaneously on Day 0, vaccination on days 7, 8 and 9, and rechallenge with MC38 cells in the opposing flank on day 70. (b) Tumour growth rate and (c) overall survival following primary challenge and rechallenge of mice vaccinated with T.VP60, S.VP60 and TS.VP60 in comparison to CPBS and VP60. An age-matched naïve population of C57BL/6 mice was used as a rechallenge control group. Statistical analysis performed using Mantel-Cox log-rank tests for Kaplan-Meier survival curve. NS = Non-significant, * p < 0.05. (JPEG 291 kb) [file 40425_2017_270_MOESM7_ESM.jpg]
